# When Salmonella Manifests Through the Skin: Erythema Nodosum in a Pediatric Case

**DOI:** 10.7759/cureus.61594

**Published:** 2024-06-03

**Authors:** Madiha Benhachem, Amal Hamami, Aziza Elouali, Abdeladim Babakhouya, Maria Rkain

**Affiliations:** 1 Department of Pediatrics, Mohammed VI University Hospital, Faculty of Medicine and Pharmacy, Mohammed First University, Oujda, MAR; 2 Department of Pediatric Gastroenterology, Centre Hospitalier Universitaire (CHU) Mohammed VI, Oujda, MAR

**Keywords:** salmonella, diarrhea, child, septal hypodermitis, erythema nodosum

## Abstract

Erythema nodosum (EN) is a non-specific nodular dermo-hypodermic rash characterized by the sudden occurrence of painful lumps located especially in the legs following a non-specific reaction to different internal and external antigens. Clinical and histological manifestations are stereotyped, regardless of the etiology. Erythema nodosum is most frequently associated with infections, particularly bacterial and less commonly viral, fungal, and parasitic. Other conditions can be discussed, including systemic diseases, malignant tumors, medicines, and vaccines. In almost half of cases, erythema nodosum is idiopathic if no cause is found. We report a case of erythema nodosum secondary to a *Salmonella* infection in a seven-year-old male. The peculiarity of our observation is the initial presentation of systemic signs that preceded the gastrointestinal symptoms.

## Introduction

Erythema nodosum (EN) is a septal hypodermitis without vasculitis characterized by the presence of lumps affecting the legs. Its positive diagnosis is essentially clinical [1,2]. Its etiologies are numerous, hence the interest of a thorough etiological investigation. We report the case of a seven-year-old male who consulted for the management of subcutaneous nodules, suggesting erythema nodosum, caused by a *Salmonella* infection.

## Case presentation

A seven-year-old child with no substantial medical background was admitted for the management of a nodular rash associated with arthralgia, evolving 10 days before admission. The clinical examination at admission found the child conscious, apyretic, and hemodynamically and respiratorily stable.

**Figure 1:**
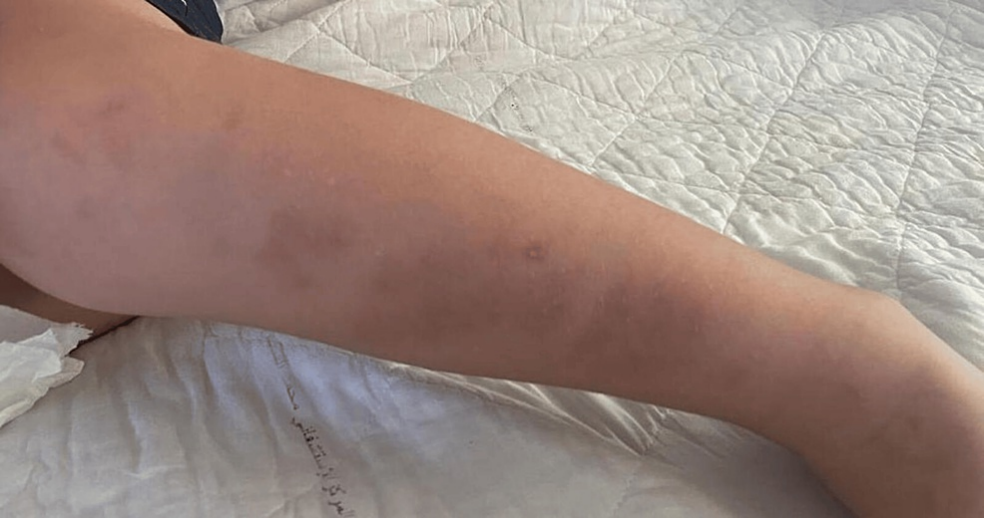
Nodular lesions visible on the legs

The skin examination found diffuse subcutaneous nodules and lumps in both legs, painful and hot on palpation (Figure 1). Gonalgia was objectified by the osteoarticular examination to both passive and dynamic knee mobilization. The remainder of the checkup was normal.

The inflammatory evaluation was positive (Table 1), viral serologies were negative, and physiological assessment was negative.

**Table 1:** Our patient's biological findings

Laboratory parameter	Test results	Reference range
Hemoglobin (g/dL)	12.4	11-13.5
White blood cell (/µL)	17,650	4,000-10,000
Lymphocyte (/µL)	2,790	2,000-4,000
Neutrophil (/µL)	13,550	1,500-7,000
Platelets (/µL)	423,000	150,000-400,000
C-reactive protein (mg/L)	100.35	<5
Sedimentation speed (mm/hour)	85	<20
Fibrinogen (g/L)	4.2	2-4
Ferritinemia (ng/mL)	107.4	15-150
Procalcitonin (ng/mL)	0.05	<0.5

The skin biopsy confirmed septal involvement without vascular involvement. Four days after being admitted to the hospital, the child had diarrhea with mucus stools. *Salmonella* was identified through stool coproparasitological examination. The diagnosis was erythema nodosum following *Salmonella* infection.

Therapeutic management was based on rest with raised limbs, symptomatic treatment of pain, and etiological treatment based on antibiotics; this therapeutic approach led to positive clinical outcomes.

## Discussion

Erythema nodosum is an uncommon skin disorder in children that manifests as painful red or purple lumps mainly located on the legs. Its prevalence varies depending on geographical origin and underlying cause; it is estimated at 1-5 per 100,000 [2]. It can occur at any age, rarely in children and exceptionally in babies [2,3]. In pediatrics, the sex ratio is equal to 1, in contrast to adults and adolescents, where a female predominance is evident [3,4]. The etiopathogenesis of erythema nodosum is still being debated; however, it is generally accepted that it is secondary to a non-specific skin reaction to various antigens. The causes are therefore multiple and varied, but in 23%-55% of cases, the erythema nodosum is said to be idiopathic without any cause found [4,5].

Secondary EN accounts for 47%-77% [6,4], and it is linked to a wide range of situations, including viral, bacterial, fungal, and parasitic infections, systemic disorders, malignancies, medications, vaccines, and pregnancy. Secondary EN caused by infections accounts for a considerable portion of the various secondary forms, 45.4%-71% of all EN-related disorders [7,4]. Group A β-hemolytic streptococcus infection is the most prevalent, accounting for up to 48% of cases in children [4]. In addition to group A β-hemolytic streptococcus, *Mycoplasma pneumoniae* is a common infectious agent implicated in EN. This infection is described in several pediatric studies; in the study by Aydın-Teke et al. [8] carried out on 39 children with EN, two cases of *Mycoplasma pneumoniae* (5.1%) were reported. Although the prevalence of *Mycobacterium tuberculosis*, the most prevalent causative agent of EN in the past, has significantly decreased in the present [9], it is nonetheless important to take into account in endemic countries. In cases of EN associated with gastroenteritis, *Yersinia enterocolitica* and pseudotuberculosis are the most common infectious agents [3]; *Salmonella* is rarely implicated [10].

Some cases of EN related to *Salmonella* infection have been described in the literature, with Grossman and Katz [11] reporting the first in 1984. Subsequently, a few cases were published. In 2010, Mantadakis et al. [12] described the case of an eight-year-old male who developed erythema nodosum during febrile gastroenteritis. Sota Busselo et al. [13] studied 45 erythema nodosum cases, seven of which were related to *Salmonella* enteritidis. In our case, the EN comes before the gastrointestinal signs, in contrast to most documented cases where the digestive signs come first.

It was noticed that gastrointestinal signs may be missing at the time of the first symptoms. Therefore, it is important to keep in mind that systemic signs may initially manifest a *Salmonella* infection and that digestive signs are not always the first to appear.

## Conclusions

An etiological investigation is a very important step in the management of erythema nodosum and must include a guided interrogation, a careful clinical examination, and a minimal paraclinical assessment. Skin biopsy is proposed in atypical forms based on their topography, semiology, terrain, and evolution or the existence of concomitant unusual manifestations.
